# A new mitochondrial gene order in the banded cusk-eel *Raneya brasiliensis* (Actinopterygii, Ophidiiformes)

**DOI:** 10.1080/23802359.2018.1532824

**Published:** 2018-11-21

**Authors:** Amir Fromm, Stephen D. Atkinson, Gema Alama-Bermejo, Paulyn Cartwright, Jerri L. Bartholomew, Dorothée Huchon

**Affiliations:** a School of Zoology, George S. Wise Faculty of Life Sciences, Tel Aviv University, Tel Aviv, Israel;; b Department of Microbiology, Oregon State University, Corvallis, OR, USA;; c Center for Applied Research and Technology Transference in Marine Resources Almirante Storni (CIMAS-CCT CONICET-CENPAT), San Antonio Oeste, Argentina;; d Department of Ecology and Evolutionary Biology, University of Kansas, Lawrence, KS, USA;; e The Steinhardt Museum of Natural History and National Research Center, Tel Aviv University, Tel Aviv, Israel

**Keywords:** Ophidiidae, mitogenomics, next-generation sequencing, phylogeny

## Abstract

The complete mitochondrial genome of the banded cusk-eel, *Raneya brasilensis* (Kaup, 1856), was obtained using next-generation sequencing approaches. The genome sequence was 16,881 bp and exhibited a novel gene order for a vertebrate. Specifically, the WANCY and the *nd6 – D-loop* regions were re-ordered, supporting the hypothesis that these two regions are hotspots for gene rearrangements in Actinopterygii. Phylogenetic reconstructions confirmed that *R. brasiliensis* is nested within Ophidiiformes. Mitochondrial genomes are required from additional ophidiins to determine whether the gene rearrangements that we observed are specific to the genus *Raneya* or to the subfamily Ophidiinae.

Mitochondrial (mt) gene orders are extremely conserved in vertebrates. In fish (Actinopterygii), only 35 departures from the canonical mt gene order have been described, whereas over 2000 species have been sequenced (Satoh et al. 2016). In contrast, the vertebrate sister clade – the tunicates – demonstrates extreme gene order variability, in which each of the sequenced genera presents a different gene order (Gissi et al. 2010; Rubinstein et al. 2013). Consequently, finding a new gene order in Actinopterygii is a rare event. The banded cusk-eel (*Raneya brasiliensis* [Kaup, 1856]) is a demersal fish present along the eastern coast of South America, from southern Brazil to northern Argentina. We report here a new mt gene order for this species.

The *R. brasiliensis* specimen we studied was collected in Argentina (43.374000 S 64.901944 W), as bycatch from a shrimp beam trawler. The sample has been deposited in the Invertebrate collection of Museo de La Plata, FCNyM-UNLP, Argentina, Acc. Number MLP-CRG 420. Our original aim was to characterize a myxozoan parasite of this species. DNA was extracted from myxozoan-infected tissue using a DNeasy Blood & Tissue Kit (Qiagen, Germantown, MD). A dual-indexed Illumina library was created using a Wafergen Biosystems Apollo 324 NGS Library Prep System (TakaraBio, Mountain View, CA), then paired-ended sequencing (150 bp), was performed on an Illumina HiSeq 3000 (Illumina, San Diego, CA) by the Center for Genome Research and Biocomputing of Oregon State University (USA). DNA reads were assembled using IDBA-UD as implemented in IDBA-1.1.1 (Peng et al. 2010) and the fish mt sequence was identified using BLAST searches. Reads were mapped with Geneious Pro version 9.0.5 using ‘High Sensitivity’ and mapping only paired reads which ‘map nearby’. Among the ∼189,000,000 reads obtained, the mean coverage of the fish mitogenome was computed with Geneious Pro and estimated to be ∼40,000 (SD ∼ 6000; Min = 22,740; Max = 60,423). Annotation was performed with MitoAnnotator (http://mitofish.aori.u-tokyo.ac.jp/annotation/input.html, last accessed 2017 Nov) (Iwasaki et al. 2013). The complete mt sequence of *R. brasiliensis* was submitted to the DNA databank of Japan (accession number LC341245).

The fish identification to species level was confirmed by constructing a phylogenetic tree based on *cox1* sequences, as recommended by Botero-Castro et al. (2016). All *cox1* sequences of Ophidiinae available on 7 December 2017 were downloaded from The National Center for Biotechnology Information (NCBI). Other Ophidiiforms with complete mt sequences were used as outgroups. *Cox1* sequences were aligned with MAFFT 7.308 (Katoh and Standley 2013) under the L-ins-i algorithm. A phylogenetic tree was reconstructed with RaxML 7.4.2 (Stamatakis 2006) using codon partitions under the GTRGAMMA model. Bootstrap percentages (BP) were computed using the rapid bootstrap option.

The phylogenetic position of *R. brasiliensis* among Ophidiiformes was investigated using all mt protein-coding genes encoded on the H-strand. The *nd6* gene and overlapping gene regions were discarded. Each protein-coding gene was aligned separately with MAFFT, as described above. A maximum likelihood (ML) tree was reconstructed with RaxML 7.4.2 as described above, with different model parameters for each codon partition of each protein-coding gene. In addition, a Bayesian reconstruction was performed using MrBayes 3.2.2 (Ronquist et al. 2012) for 12,500,000 generations under default mcmc settings. The partitions and substitution models were the same as those for the ML analysis.

The *R. brasiliensis* mt genome was 16,881 bp, slightly longer than other Ophidiiformes (16,090–16,564 bp). Surprisingly, we identified that the mt gene order was rearranged compared with the standard Actinopterygii gene order (Figure 1). Specifically, we observed different orders in two regions: the WANCY tRNA gene cluster and the *nd6* – *D-loop* region. All rearranged genes had retained their original strand direction, as observed in other Ophidiiformes. In *R. brasiliensis*, the *trnN* was transposed to the end of the ‘WANCY’ region, presenting a gene order of WACYN (Figure 1(A)). The exact position of the origin of light-strand replication (O_L_), which is usually located between *trnN* and *trnC* in Actinopterygii, could not be determined. Concerning rearrangement of the *nd6* – *D-loop* region, in the standard mitochondrial gene order the *cytb* gene is usually flanked by the *trnE* and *trnT* on its 5′- and 3′-ends, respectively (Figure 1(B)). In *R. brasiliensis,* the *cytb* gene was flanked by non-coding regions and the *nd6* +* trnE* gene region was transposed downstream of the *cytb* gene. The *trnE* is now flanked by the *trnP* at its 3′-end. This indicated that both *nd6* +* trnE* and *trnT* gene regions have been transposed. The *trnT* is now found downstream to the D-loop (or control region), and is flanked at its 3′-end by a pseudo-*trnP*, which suggests that the transposition of the *trnT* involved the duplication of the *trnT + trnP* region (Figure 1(A)).

**Figure 1.**
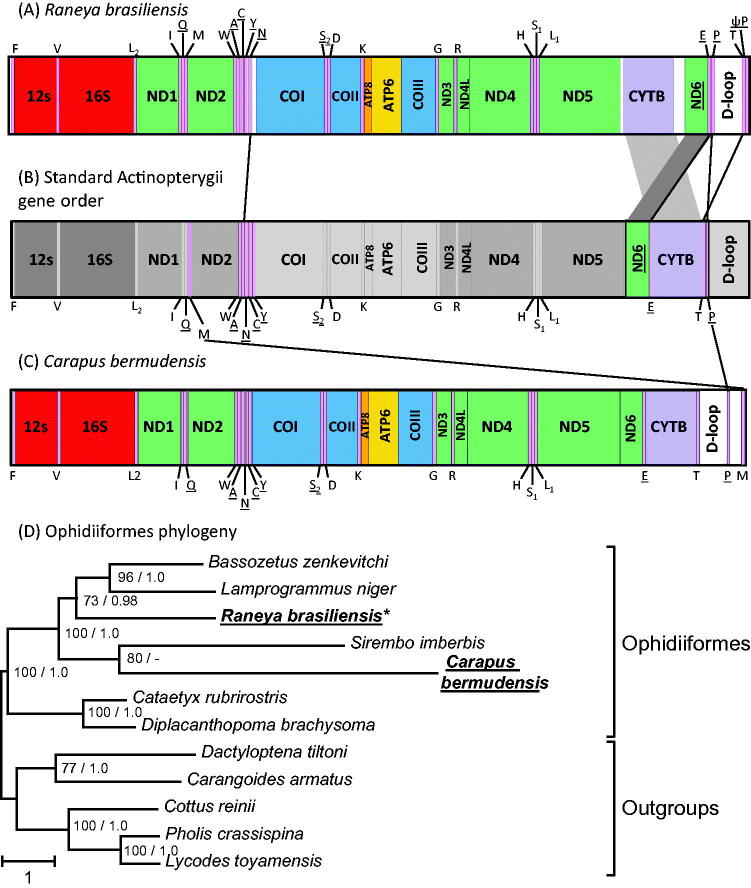
Linearized representation of *Raneya brasiliensis* mt gene order (A) compared with the typical Actinopterygii mt gene order (B) and with *Carapus bermudensis* mt gene order (C). tRNA genes are designated by single-letter amino acid codes. Genes that have undergone rearrangement in *R. brasiliensis* (A) and *C. bermudensis* (C) are connected with lines to their corresponding location in the typical Actinopterygii gene order (B). Genes encoded on the L-strand are underlined. The phylogenetic position of *R. brasiliensis* and *C. bermudensis* among Ophidiiformes was reconstructed based on mt protein-coding genes (D). All species possess the typical Actinopterygii mt gene order except *R. brasiliensis* and *C. bermudensis*, which are indicated in bold. Bootstrap supports above 50% and Bayesian posterior probabilities are indicated near the corresponding nodes, separated with a slash. The mt sequence of the specimen obtained in this work is indicated in bold and with an asterisk.

Our phylogenetic reconstruction based on *cox1* sequences (Figure 2) confirmed that the obtained sequence clusters with other *R. brasiliensis* (EU074577 and EU074578 (Mabragaña et al. 2011)) with maximal support value (BP = 100). The 652 bp *cox1* sequences of the three specimens differed by 1–2 nucleotides only, supporting the correct identification of our sample. We then investigated the position of *R. brasiliensis* within the Ophidiiformes using mt protein coding sequences (Figure 1(D)). We found that *Raneya* (Ophidiinae) was a sister clade of the Neobythitinae *Bassozetus zenkevitchi* and *Lamprogrammus niger* with high support (BP = 73; posterior probability PP = 0.98). In agreement with Miya et al. (2003), our analyses did not recover the monophyly of Ophidiidae, as *Carapus bermudensis* (Carapidae, Carapinae) is the sister clade of *Sirembo imberbis* (Neobythitinae) (Figure 1(D)).

**Figure 2.**
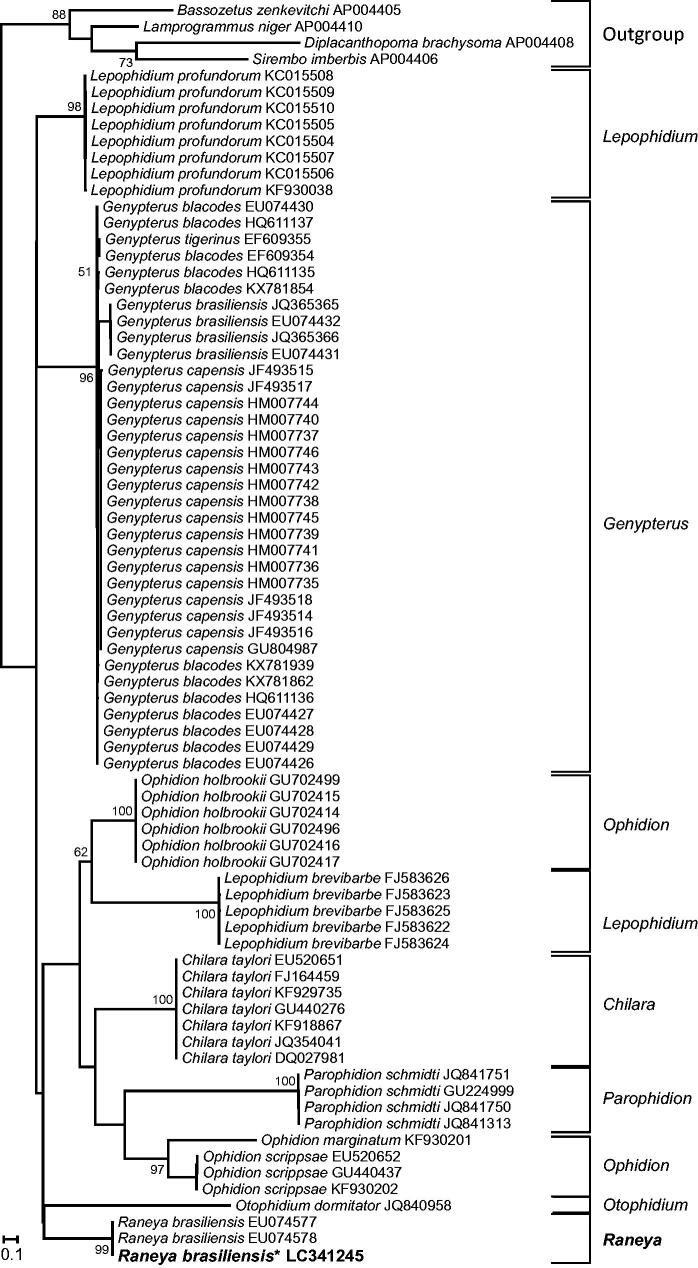
Maximum likelihood tree of Ophidiinae *cox1* sequences. The *cox1* sequence of the specimen obtained in this work is indicated in bold and with an asterisk. Bootstrap supports above 50% are indicated near the corresponding node.

Mitochondrial gene order is highly conserved among vertebrates, thus finding a novel rearrangement is a rare event. In this study, we identified multiple unique rearrangements in *R. brasiliensis*, a representative of the Ophidiiformes. Interestingly mt rearrangements have been described in another member of the Ophidiiformes – *C. bermudensis* (Miya et al. 2003; Satoh et al. 2016). However, the rearrangements in *Carapus* and *Raneya* differ. In *Carapus,* they involve the *trnP* and *trnM*, which are located downstream of the D-loop (Figure 1(C)). Our phylogenetic analyses (Figure 1(D)) showed that *Carapus* and *Raneya* are not closely related, and are both more closely related to species that have a standard Actinopterygii gene order. These findings support the hypotheses that mt rearrangements occurred independently in *Carapus* and *Raneya*, and that the Ophidiiformes constitute a hotspot for gene rearrangement.

Gene rearrangements in Actinopterygii occur more frequently in the WANCY and the region from the *nd5* to the D-loop (Satoh et al. 2016). Our results support this view as the *R. brasiliensis* rearrangements occurred in these specific regions. Tandem duplications followed by random loss is the favoured model to explain mt rearrangements in vertebrates (Satoh et al. 2016). Our finding of a duplicated pseudo-*trnP* gene in *R. brasiliensis* supports this view. However, the tandem duplication-random loss model would require at least three separate events of duplication with multiple gene losses, in the lineage leading to *Raneya.* Additional sequencing of members of the Ophidiinae should shed light on the origins of the novel *Raneya* gene order. Additional data should also reveal whether the gene order we observed in *Raneya* is shared by other members of the Ophidinae or whether it is specific to *Raneya*.
